# Treatment planning and 4D robust evaluation strategy for proton therapy of lung tumors with large motion amplitude

**DOI:** 10.1002/mp.15067

**Published:** 2021-07-17

**Authors:** Vicki Trier Taasti, Djoya Hattu, Femke Vaassen, Richard Canters, Marije Velders, Jolein Mannens, Judith van Loon, Ilaria Rinaldi, Mirko Unipan, Wouter van Elmpt

**Affiliations:** ^1^ Department of Radiation Oncology (MAASTRO) GROW – School for Oncology Maastricht University Medical Centre+ Maastricht Netherlands

**Keywords:** large tumor amplitude, lung tumors, Proton treatment planning, robust evaluation, 4DCT evaluation

## Abstract

**Purpose:**

Intensity‐modulated proton therapy (IMPT) for lung tumors with a large tumor movement is challenging due to loss of robustness in the target coverage. Often an upper cut‐off at 5‐mm tumor movement is used for proton patient selection. In this study, we propose (1) a robust and easily implementable treatment planning strategy for lung tumors with a movement larger than 5 mm, and (2) a four‐dimensional computed tomography (4DCT) robust evaluation strategy for evaluating the dose distribution on the breathing phases.

**Materials and methods:**

We created a treatment planning strategy based on the internal target volume (ITV) concept (aim 1). The ITV was created as a union of the clinical target volumes (CTVs) on the eight 4DCT phases. The ITV expanded by 2 mm was the target during robust optimization on the average CT (avgCT). The clinical plan acceptability was judged based on a robust evaluation, computing the voxel‐wise min and max (VWmin/max) doses over 28 error scenarios (range and setup errors) on the avgCT. The plans were created in RayStation (RaySearch Laboratories, Stockholm, Sweden) using a Monte Carlo dose engine, commissioned for our Mevion S250i Hyperscan system (Mevion Medical Systems, Littleton, MA, USA). We developed a new 4D robust evaluation approach (4DRobAvg; aim 2). The 28 scenario doses were computed on each individual 4DCT phase. For each scenario, the dose distributions on the individual phases were deformed to the reference phase and combined to a weighted sum, resulting in 28 weighted sum scenario dose distributions. From these 28 scenario doses, VWmin/max doses were computed. This new 4D robust evaluation was compared to two simpler 4D evaluation strategies: re‐computing the nominal plan on each individual 4DCT phase (4DNom) and computing the robust VWmin/max doses on each individual phase (4DRobInd). The treatment planning and dose evaluation strategies were evaluated for 16 lung cancer patients with tumor movement of 4–26 mm.

**Results:**

The ratio of the ITV and CTV volumes increased linearly with the tumor amplitude, with an average ratio of 1.4. Despite large ITV volumes, a clinically acceptable plan fulfilling all target and organ at risk (OAR) constraints was feasible for all patients. The 4DNom and 4DRobInd evaluation strategies were found to under‐ or overestimate the dosimetric effect of the tumor movement, respectively. 4DRobInd showed target underdosage for five patients, not observed in the robust evaluation on the avgCT or in 4DRobAvg. The accuracy of dose deformation used in 4DRobAvg was quantified and found acceptable, with differences for the dose‐volume parameters below 1 Gy in most cases.

**Conclusion:**

The proposed ITV‐based planning strategy on the avgCT was found to be a clinically feasible approach with adequate tumor coverage and no OAR overdosage even for large tumor movement. The new proposed 4D robust evaluation, 4DRobAvg, was shown to give an easily interpretable understanding of the effect of respiratory motion dose distribution, and to give an accurate estimate of the dose delivered in the different breathing phases.

## 1 INTRODUCTION

Intensity‐modulated proton therapy (IMPT) has shown to deliver lower doses to the heart as well as the individual substructures of the heart compared to volumetric modulated arc photon therapy (VMAT) for locally advanced lung cancer.^1^ A reduction in mean heart dose can lower the risk of mortality, especially for lung patients with a small tumor volume,^2^ whereby IMPT might be beneficial for these patients. Compared with passive‐scattering proton therapy (PSPT), IMPT has also been shown to reduce the mean dose to the lungs.^3^ However, IMPT is more sensitive to changes in the beam path than PSPT and photon therapy, which makes IMPT of lung patients more challenging due to the breathing motion. The changes in the beam path during the breathing cycle could change the proton range and lead to suboptimal coverage of the tumor for large tumor movement.^4^, ^5^

In the AAPM guidelines on motion management for tumors affected by respiratory motion, such as lung tumors, respiratory motion management is recommended for tumors with a movement larger than 5 mm.^6^ It has, moreover, been found that target underdosage, if not explicitly accounted for, was significantly higher for patients with a tumor movement larger than 5 mm.^7^ Following the AAPM recommendations and the criterion used at other clinics,^3^ patients with a tumor movement above 5 mm are not routinely treated with IMPT. Several technological solutions have been suggested to ensure dose coverage of the tumor despite the movement, including repainting,^8^ gating,^9^ and breath hold.^10^, ^11^ However, these are technologically demanding, and techniques as breath hold also rely on patient compliance.^12^

To increase the number of patients benefitting from proton therapy, a treatment planning strategy for lung cancer patients with tumor movement larger than 5 mm needs to be established. One of the least cumbersome solutions for ensuring full target coverage for moving tumors is to use an internal target volume (ITV) structure which encompasses the whole region where the clinical target volume (CTV) can be located throughout the breathing cycle.^13^, ^14^ Increasing the spot size is another simple strategy suggested for mitigating the impact of tumor motion.^15^ The spot size can be increased by using a range shifter, but some proton delivery systems have an inherently larger spot size, including the compact proton therapy system Mevion S250i Hyperscan (Mevion Medical Systems, Littleton, MA, USA).^16^

Several evaluation strategies have been suggested for assessing if full target coverage is ensured despite tumor movement. Often these methods are based on four‐dimensional computed tomography (4DCT). Ribeiro et al. proposed a 4D robust evaluation strategy which accounts for robustness (i.e., setup and range uncertainty), breathing motion by using 4DCTs, interplay effect assessed from the treatment machine log files, and anatomical changes from the weekly repeat 4DCTs.^17^ This comprehensive approach is very elaborately evaluating all types of errors at the same time, and it assesses the full accumulated dose actually delivered over the full course of treatment. This dose can, however, only be evaluated at the end of treatment course. Up‐front evaluation if the created plan can be safely delivered to the patient needs to be performed in a different way. Another 4DCT‐based evaluation approach has been proposed by Souris et al.^18^ Their method does not take anatomical changes into account, as only the planning 4DCT is applied; however, fractionation is considered by simulating each treatment fraction individually. Moreover, they apply a realistic model for range and setup errors by using Monte Carlo sampling of the error scenarios; it was found that 300 sampled error scenarios had to be included.^18^

We introduced an easily implementable and robust treatment planning approach for proton treatment of lung cancer patients with tumor movement above 5 mm. To avoid increasing treatment planning complexity, we propose a free‐breathing treatment based on the ITV concept. In this study, we investigated the ITV‐based treatment planning strategy combined with the spot size characteristics of a compact sized proton therapy machine. Moreover, to ensure target coverage despite the large tumor movement, we developed a 4D evaluation strategy to assess the dose to the target and the organs at risk (OARs) throughout the breathing cycle. Our 4D evaluation strategy was inspired by the strategies proposed by Ribeiro et al.^17^ and Souris et al.^18^ However, we did not include repeated 4DCT scans or advanced error sampling, as we wanted an evaluation strategy to assess the effect of tumor motion before the start of treatment that allows to check that the plan was robust toward motion and therefore safe to deliver.

## 2 MATERIALS AND METHODS

### 2.1 Treatment planning strategy

Sixteen lung cancer patients, who had previously been treated with photon therapy at our clinic, were included in this study. The patients were selected to have large tumor motion: the tumor movement was between 4 and 26 mm. Institutional review board approval was granted for this study. Patient specifications can be seen in Table 1.

**TABLE 1:** Patient specifications including tumor location, primary target volumes (GTVp, CTVp, and ITVp), tumor amplitude (specified as the 1D distance between the midpoints of the GTVp on the two extreme phases), and the direction of the largest tumor movement, as well as the two extreme phases

Patient no.	Tumor location	GTVp volume on CT50ex (average over the 8 phases) (cm^3^)	CTVp volume on CT50ex (average over the 8 phases) (cm^3^)	ITVp volume on avgCT (cm^3^)	Amplitude (mm) (direction)	Extreme phases
1	LLL	10.9 (12.0)	31.5 (33.6)	46.9	10.1 (SI)	CT0in; CT75ex
2	RMLL	89.8 (91.1)	169.7 (169.7)	214.6	6.8 (SI)	CT0in; CT100in
3	RLL	46.2 (45.1)	87.7 (84.1)	115.2	13.4 (SI)	CT0in; CT100in
4	RLL	12.6 (12.7)	37.3 (34.3)	51.7	10.7 (SI)	CT0in; CT100in
5	LLL	20.5 (20.7)	44.7 (44.9)	62.6	8.6 (SI)	CT50in; CT75ex
6	LLL	20.4 (21.2)	51.1 (49.7)	79.7	16.0 (SI)	CT25in; CT50ex
7	RLL	22.9 (23.4)	56.1 (58.1)	92.0	14.7 (SI)	CT0in; CT75in
8	RLL	79.5 (82.1)	161.9 (163.3)	213.1	9.0 (SI)	CT0in; CT100in
9	RMLL	525.3 (511.0)	760.2 (738.3)	897.8	12.1 (SI)	CT0in; CT100in
10	LUL	35.8 (36.0)	85.6 (81.4)	94.4	3.5 (AP)	CT0in; CT100in
11	LUL	8.4 (8.7)	26.2 (27.1)	33.2	3.8 (SI)	CT0in; CT75in
12	RLL	12.4 (12.2)	38.9 (35.3)	78.8	26.1 (SI)	CT0in; CT100in
13	LUL	11.5 (11.8)	32.0 (31.4)	46.8	9.1 (SI)	CT0in; CT75in
14	RLL	10.2 (10.3)	33.8 (33.8)	50.0	7.0 (SI)	CT0in; CT100in
15	LLL	6.3 (6.7)	20.1 (19.3)	28.6	11.0 (AP)	CT0in; CT100in
16	LLL	48.5 (49.6)	96.6 (95.0)	140.8	16.1 (SI)	CT0in; CT100in

Abbreviations: AP, anterior‐posterior; LLL, left lower lobe, LUL, left upper lobe; RLL, right lower lobe; RMLL, right middle/lower lobe; SI, superior‐inferior.

Each patient had a treatment planning 4DCT scan (Siemens Drive or Confidence CT scanner, Siemens Healthineers, Forchheim, Germany). For most patients, the 4DCT resolution was 0.98 × 0.98 × 3 mm^3^, but for two patients the resolution was 0.98 × 0.98 × 1 mm^3^ and 1.27 × 1.27 × 3 mm^3^, respectively. The respiratory signal was recorded using an Anzai pressure belt (Anzai Medical Systems, Tokyo, Japan) and the 4DCT was reconstructed into eight phases using amplitude binning. The OAR and the gross tumor volume (GTV) structures were delineated on the 50% expiration CT phase (CT50ex), selected as the reference phase of the 4DCT scan. The delineated structures were propagated to the other phases using deformable image registration in RayStation based on the ANACONDA model.^19^ The default settings and no focus or controlling regions of interest (ROIs) were used. The propagated contours for the primary GTV (GTVp) on the seven other phases of the 4DCT were checked by an experienced lung radiation oncologist and adjusted if needed. The CTVs for both the primary tumor (CTVp) and the nodes (CTVn) were created as a 5‐mm expansion of the respective GTVs. Adjustments of the CTVs to anatomical structures, such as major vessels and bones, were performed manually by the physician on each 4DCT phase and on each CT slice.

The tumor movement was defined as the largest difference between the midpoint positions for the GTVp on any two phases of the 4DCT along one of the three anatomical directions. The two phases with the largest distance were denoted the extreme phases. The amplitude, the extreme phases, and the direction of largest movement are listed in Table 1.

To take motion of the tumor into account during plan optimization, an ITV (denoted ITVp) was created as the union of the CTVp structures on the eight phases. An expanded contour, ITVp_02, was created as a 2‐mm isotropic expansion of the ITVp structure to take, amongst others, the delineation uncertainty into account and as an additional safety margin to be robust against possible anatomical changes during treatment. No target CT number override was used during treatment planning. The treatment plan was optimized on the average CT (avgCT), created as an average of all eight phases of the 4DCT. It was hypothesized that the target coverage for the ITVp_02 on the avgCT would ensure dose coverage for the individual CTVp in each breathing phase.

For plan optimization, all contours (targets and OARs) were rigidly copied from the CT50ex to the avgCT (see representative example in Figure S1 in the Supplementary Material (SM)). The plans were robustly optimized by applying 3D robust optimization with our clinically used robustness settings with an isotropic setup shift of 5 mm and density uncertainty of 3%. We applied the setup uncertainty to the full dose distribution and not per individual beam, leading to 21 scenarios being included in the robust optimization (one nominal (i.e., with no included errors), six setup shifts along the axes, two range uncertainties, and 12 combined setup and range uncertainties). The RayStation robust optimization approach is based on minimax optimization.^20^, ^21^ Robust objectives were used for the target structures (ITVp_02 and CTVn) and the spinal cord. For the rest of the OARs, only the nominal scenario was considered during the optimization. All plans were optimized to a prescription dose of 60 Gy (RBE; relative biological effectiveness, with a constant scaling factor of 1.1) in 30 fractions. We did not apply 4D robust optimization, as this would increase the optimization time^22^ due to the increased number of error scenarios (168 scenarios instead of 21, when including all eight 4DCT phases). To aim for a clinically feasible treatment planning strategy, we investigated if the 3D robust optimization was sufficient to ensure full target coverage over the full breathing cycle.

The untrimmed spot size in air at the iso‐center ranges from 4.1 mm at 227 MeV to 16.4 mm at 45 MeV for our Mevion S250i Hyperscan system.^16^ However, field collimation is reached through a motorized adaptive aperture to shape the outer border of the treatment field in each energy layer, similar to a multi‐leaf collimator (MLC) in a conventional photon linear accelerator. The spot size can be trimmed by up to 50% using the MLCs, and thereby reducing the dose to surrounding healthy tissue.^23^ The treatment plans were created in RayStation 9B or 10A (RaySearch Laboratories, Stockholm, Sweden). Monte Carlo dose calculations were used both for optimization and final dose calculation, as well as for all dose evaluations, to take the MLCs into account. The Monte Carlo uncertainty for the dose computations was 1%, and 5000 protons per spot were simulated. The dose grid was 0.3 × 0.3 × 0.3 cm^3^. The beam arrangements consisted three or four beams, except for patient 9 who had a large ITVp volume, here a double iso‐centric beam configuration with six beams was used. Typically, one anterior beam, one posterior beam, and one posterior oblique beam were used, but the beam angles were tailored to each individual patient.

### 2.2 3D robust evaluation on average CT

Consistent with Dutch national consensus on proton planning, the acceptability of the plans was judged based on robust plan evaluation on the treatment planning CT (pCT), which is the avgCT for lung patients. The voxel‐wise minimum (VWmin) and maximum (VWmax) dose distributions were computed over 28 error scenarios (14 setup shifts of 5 mm combined with two density changes of ±3%),^24^ that is, the sets of error scenarios included in the robust evaluation and in the robust optimization differed, though with some overlap.

The clinical constraints used to evaluate the acceptability of proton lung plans included D95%>95% of the prescription dose (D_pres_) and V95%>95% for the target structures (ITVp_02 and CTVn) for the VWmin dose distribution; D0.03cc <76 Gy for heart, esophagus and mediastinal envelope expanded by 5 mm (MedEnv_05), and D0.03cc <54 Gy for spinal cord for the VWmax dose distribution; V5Gy <60% for contralateral lung and mean dose <20 Gy for the total lung excluding the GTV for the nominal dose; mean heart dose and mean esophagus dose for the nominal dose should preferably also be lower than 10 and 26 Gy, respectively.

### 2.3 4D robust evaluation

The standard 3D robust evaluation, described above, does not take movement into account. The proton path length will change when the tumor moves through the low‐density lung tissue, and the proton path length can also change if surrounding anatomical structures move within the beam path with the breathing; for example, the diaphragm or cardiac movement. Therefore, we wanted to evaluate if our proposed treatment planning strategy was robust during a free breathing treatment, that is, if the tumor was also covered adequately considering the entire breathing cycle. As we have a fractionated treatment with up to 30 fractions, we did not take interplay effects into account.

We developed a new 4D robust evaluation strategy, which takes the time spend in each breathing phase into account. We compared this strategy to two other 4D evaluation strategies to show that such simple strategies do not accurately take motion into account.

#### 2.3.1 4D robust phase‐averaged evaluation (4DRobAvg)

**FIGURE 1:**
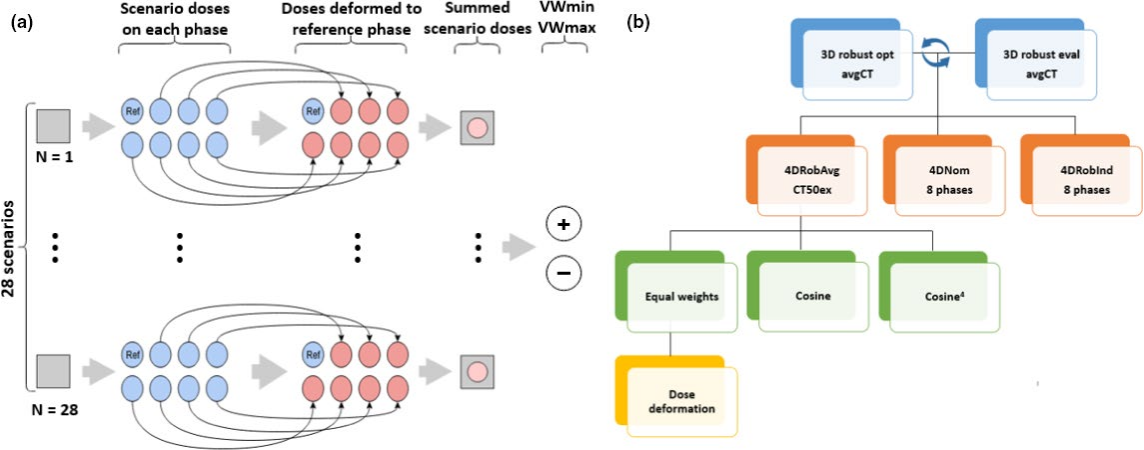
(a) The steps in the 4DRobAvg evaluation strategy (see Section 2.3.1). (b) Overview of the comparisons performed in this study

We developed a new 4D evaluation strategy which will be denoted 4D robust phase‐averaged evaluation (4DRobAvg). In this strategy, each of the 28 robust evaluation scenario doses were computed on all eight 4DCT phases. Using deformable image registration, the doses from the individual phases were warped to the reference phase (in our case CT50ex). For each of the 28 robustness scenarios, a weighted sum was computed over the eight scenario doses from the individual phases, to account for the time spent in each phase. From the 28 weighted sum scenario doses, the composite VWmin and VWmax dose distributions were computed. The main steps are as follows (Figure 1a):

Loop over the error scenarios (28 in our case). In each scenario:

1. Compute the scenario dose on each of the individual 4DCT phases.
2. Propagate the dose from each of the individual phases to the reference phase by deformable image registration.
3. Compute the weighted sum of eight doses on the reference phase.

Compute the composite VWmin and VWmax dose distributions based on the 28 weighted sum scenario dose distributions.

The weighted sum for the nominal dose computed on each phase and deformed to the reference phase was also computed. In total, the 4DRobAvg evaluation outputs three dose distributions: (1) The nominal weighted dose, (2) the VWmin, and (3) the VWmax for the weighted sums over the scenario doses.

For the dose warping in step 2, the same deformable image registrations were used as for the contour propagation described in Section 2.1.

Since the exact breathing pattern for a patient is not known, we assumed that the tumor will spent an equal amount of time in each phase by using equal weights for each phase (w = 1/8 in our cases as we had eight phases) in the weighted sum in step 3. This assumption was, however, not always valid in our case, since we used an amplitude‐binned 4DCT reconstruction and not phase‐binned 4DCT reconstruction. To evaluate the size of the introduced error, we tested two other weighting schemes, which assumed the breathing signal to follow either a cosine function or a cosine to the fourth power (cos^4^). The weights used for these two breathing patterns can be seen in Figure S2 in SM. We compared the dose‐volume histogram (DVH) parameters obtained for the three breathing signals. We did not evaluate the effect of breathing pattern changes.

#### 2.3.2 4D nominal evaluation (4Dnom)

A simple 4D evaluation strategy is to re‐compute the nominal dose on each of the eight phases of the 4DCT. This 4D evaluation strategy will be denoted 4D nominal evaluation (4DNom). When presenting the results for the 4DNom strategy, the worst value was taken for each DVH parameter, that is, the lowest target dose or the highest OAR dose over the eight phases.

#### 2.3.3 4D robust individual phase evaluation (4DRobInd)

To keep the simplicity, but take setup and range robustness into account, a 3D robust evaluation can be performed on each phase separately. This strategy will be denoted 4D robust individual phase evaluation (4DRobInd). Using the same 3D robust evaluation as described in Section 2.2, this strategy will result in eight VWmin and eight VWmax dose distributions. For this strategy, we will present the average value for the DVH parameters over the eight phases. The important difference between 4DRobInd and 4DRobAvg is that 4DRobInd averages over the eight VWmin/VWmax dose distributions whereas 4DRobAvg averages the breathing phases and then creates one worst‐case dose distribution based on these phase‐averaged dose distributions.

### 2.4 Evaluation of dose deformation

The 4DRobAvg strategy is based on the assumption that the dose propagation is physically sound. As this assumption is a very crucial part of the evaluation, we incorporated a check of the dose differences before and after the dose deformation. The DVH parameters were extracted both before and after deforming the dose toward the reference phase. Before deforming the dose distribution, the DVH parameters were extracted from the contours on the original phase, and after deforming the dose to the reference phase, the DVH parameters were extracted from the contour set on the reference phase. Therefore, both the contour set and the dose distribution differed between the DVH parameters extracted before and after deforming the dose, assuming that the dose deposited to a given ROI on one phase would be deformed to the location of this ROI on the reference phase, since the structures and the dose were deformed using the same deformable image registration (see Figure 3 below). We assumed dose differences larger than 1 Gy to be of clinical relevance, considering that the prescription dose was 60 Gy. We presented the average and the maximum dose differences over the 29 scenarios (28 robustness scenarios and the nominal scenario) for each of the seven non‐reference phases. These results are only shown for the breathing pattern assuming equal weights.

### 2.5 Evaluation strategy

In Figure 1b, the different evaluations performed in this study are presented. 3D robust optimization and 3D robust evaluation were performed iteratively until a clinical plan was obtained (top row). The 4D evaluations were performed on the final plan (second row). For the 4DRobAvg evaluation, the assumption of the weighting scheme used for combining the doses on the individual phases is investigated (third row), and for the equal weight scheme, the accuracy of the dose deformations between the phases was also evaluated (fourth row).

The results presented for the target structures were extracted from the VWmin dose distribution, and for OARs with a maximum dose constraint (quantified as the D0.03cc), the results were extracted from the VWmax dose distribution. For OARs with a mean dose constraint, results were evaluated from the nominal dose distribution.

## 3 RESULTS

For all 16 patients, it was possible to create a plan which satisfied all clinical constraints for the dose distribution on the planning CT (avgCT). Despite large tumor motion up to 26 mm, no OAR overdosage was seen for any of the patients. The large tumor motion resulted in large target volumes (Table 1) due to the use of the ITV concept. To quantify the effect of the ITV approach on the target size, we calculated the ratio of ITVp volume to the average CTVp volume over the eight phases. This ratio increased approximately linearly with the amplitude size (Figure S3, SM), with an average ratio of 1.4 over the 16 patients. Moreover, we compared the primary target volume used for robust optimization, ITVp_02, with the primary target used for non‐robust photon optimization, PTVp (primary planning target volume).

### 3.1 4D robust plan evaluation

#### 3.1.1 Timing

For the two robust 4D evaluation methods, 4DRobAvg and 4DRobInd, almost the same computations are performed and therefore the timing of the two methods are similar; in median, the 4DRobAvg takes 0.85 h (range: [0.61, 1.92] h), and 4DRobInd takes 0.71 h. The small extra time needed for 4DRobAvg is due to the dose deformations, weighted dose summations, and the nominal dose computation on the phases to create a weighted sum for the nominal scenario as well. For only two patients, the computation time was above 1 h; the longest computation time (1.92 h) is seen for patient 9 who had six treatment beams. The timing of the 4DNom is much shorter, in median only 62 s, since no robustness scenarios are computed.

#### 3.1.2 Target dose

**FIGURE 2:**
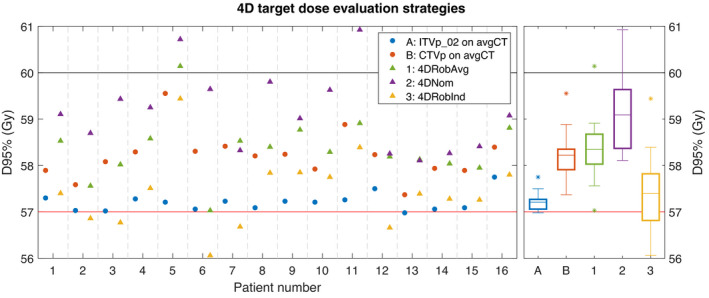
D95% for the primary target in the three‐dimensional (3D) robust evaluation and the different four‐dimensional (4D) evaluation strategies. The results for the individual patients are shown at the left and boxplots over the 16 patients at the right. The horizontal black line indicates the prescribed dose, and the horizontal red line indicates the minimum dose constraint. For the boxplots, the outliers (star markers) are defined as data points more than 1.5 times the interquartile range outside the box. *Legend explanation*: A and B: 3D robust evaluation results on the planning CT (avgCT); 1–3: 4D evaluation strategies for CTVp. For the robust evaluations (A, B, 1, and 3), the results are given for the VWmin evaluations

In Figure 2, the different 4D dose evaluation strategies were compared based on the primary target, CTVp. For comparison, we also included the results for the 3D robust evaluation, both for ITVp_02 and CTVp. A single patient (patient 13) had a D95% = 56.98 Gy; this minor underdosage was accepted. For all patients, the 4DNom resulted in the highest D95% values, except for patient 7, where the 4DNom result on a single phase was 0.1 Gy lower than the VWmin on the avgCT.

The average D95% for the 4DRobInd fell below the dose constraint for five patients. Four of these five patients had an overlap of the ITVp with the diaphragm on at least one phase. Looking at the individual values for the VWmin D95% on the individual phases, 11 out of 16 patients had one or more phases failing the dose constraint (results not shown).

**FIGURE 3:**
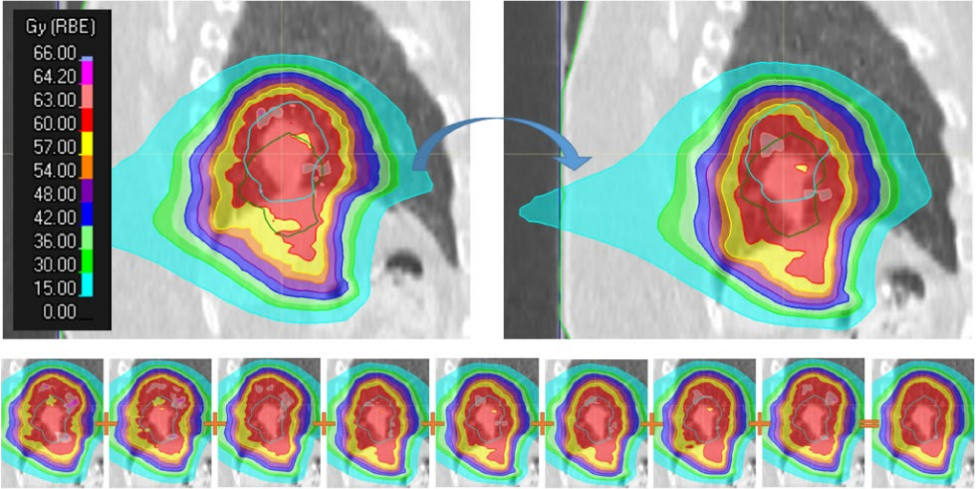
Top: Illustration of the dose deformation from the original (non‐reference) phase (top left) to the reference phase (top right). In both images, the light blue delineation is the CTVp on the original phase, while the green delineation is the CTVp on the reference phase. In this example, the dose is correctly deformed from the position of the CTVp on the original phase to the position of the CTVp on the reference phase. Bottom: Sum of the dose on the reference phase and the seven doses deformed to the reference phase. Areas of under‐ and overdosage are seen for each individual dose distribution (first eight images), but the weighted sum dose (rightmost figure; applying equal weights) is homogeneous and no under‐ and overdosage is seen in the area of the CTVp (light blue delineation)

The results for 4DRobAvg fulfilled the dose constraint for all patients, even though the dose on the individual phases (4DRobInd) failed for 11 patients. In Figure 3, an example of the dose deformation and weighted sum formation is illustrated. In this example, the under‐ and overdosage areas are averaged out during the breathing cycle. This illustrates the importance of taking the time spent in each phase into account.

#### 3.1.3 Organ at risk doses

**FIGURE 4:**
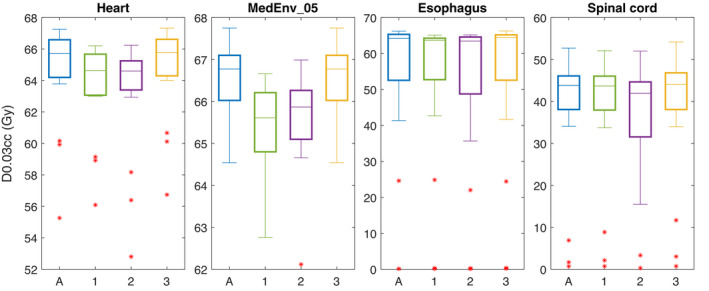
D0.03cc in the four‐dimensional (4D) evaluation strategies. A: VWmax for 3D robust evaluation on avgCT. 4D evaluation strategies: 1: 4DRobAvg, 2: 4DNom, 3: 4DRobInd

The results for the comparison among the three 4D evaluation strategies on the OARs are shown in Figure 4 for the four OARs with a maximum dose constraint. The results are similar for the four evaluations, the 3D evaluation on the avgCT and three 4D evaluation strategies. Patients 6 and 7 had no nodal targets, for these patients the esophagus dose was around 0 Gy. The differences in mean dose for the heart, esophagus and lungs extracted from the nominal dose on the avgCT, the 4DNom and the 4DRobAvg nominal weighted sum were minor; the median over the patients of the three evaluation was less than 1 Gy for all three OARs (results not shown).

### 3.2 Weighting schemes for 4DRobAvg

**FIGURE 5:**
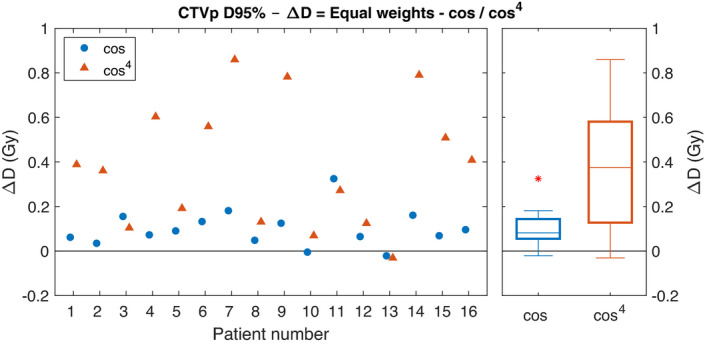
Differences for D95% for CTVp between applying equal weights and either a cos or a cos^4^ breathing pattern. At the left, the results for the individual patients, and to the right, the boxplots over all 16 patients

**FIGURE 6:**
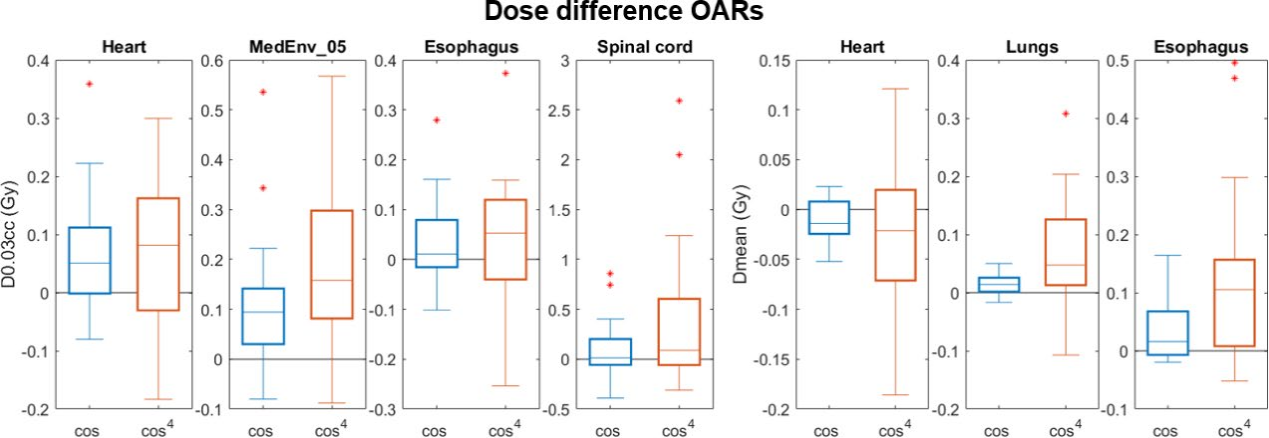
Dose differences between equal weights and a cos or a cos^4^ breathing pattern. The four leftmost subplots show the dose differences for D0.03cc for the VWmax dose distribution, while the three rightmost subplots show the difference in the mean dose for the nominal doses. Note the different y‐axis ranges

Only small differences are seen for the DVH parameters between the weighting schemes used in the 4DRobAvg applying equal weights and cosine weights, with a median difference of 0.1 Gy for the CTVp D95% (Figure 5). Larger differences were seen between equal weights and cos^4^ weights. Here, the median and maximum differences for CTVp D95% were 0.4 and 0.9 Gy, respectively (Figure 5). For the spinal cord, the largest difference for D0.03cc was 2.6 Gy; however, the median difference was 0.1 Gy. For the rest of the OAR DVH parameters, smaller differences were seen (Figure 6).

### 3.3 Evaluation of dose deformation

The Dice scores for the deformed GTVp delineations between the unadjusted and the adjusted contours were calculated for the seven propagated contours on the non‐reference phases. For 54% of the contours, the Dice score was above 0.95, while another 40% was between 0.90 and 0.95. The lowest Dice score of 0.79 was found for patient 12, who had an amplitude of 26 mm. But in general, no correlation was seen between Dice score and the distance the contour was propagated (Figure S4, SM).

For the dose deformation test, the difference in the DVH parameters before and after deforming the dose from the original phase toward the reference phase is shown in Figure S5 (SM) for the D95% for the CTVp and for D0.03cc for the esophagus which showed the largest differences. For the CTVp D95%, only two patients (7 and 14) exceeded the threshold of 1 Gy, with maximum dose differences over the 29 scenarios of 1.9 and 1.5 Gy, respectively. For the D0.03cc to the esophagus, the maximum DVH difference exceeded 1 Gy for more patients for one or more breathing phases, with differences up to 4.6 Gy. The differences for the rest of the DVH parameters were typically below the 1 Gy threshold.

## 4 DISCUSSION

We have evaluated a new treatment planning strategy for lung tumors with a large motion. A clinically acceptable plan with full target coverage and no OAR overdosage could be created for all 16 patients, even though the tumor movement for these patients was up to 26 mm. To evaluate the effect of breathing motion on the dose distribution, we developed and tested a new 4D robust evaluation strategy, 4DRobAvg. This new evaluation strategy accounted for both motion due to breathing and setup and range errors.

In this study, three 4D evaluation strategies were compared. In the 4DRobInd scheme, the robustness is evaluated on each phase separately. However, range and setup errors are systematic over one fraction; therefore, the different phases need to be treated in combination for each error scenario. As shown in Figure 4, the under‐ and overdosage might be located differently in the individual 4DCT phases, and this can be exaggerated by computed the VWmin and VWmax separately for each phase, as in 4DRobInd. Instead, the cold and hot spots might average out over the breathing cycle. The 4DRobAvg evaluation strategy takes this into account by performing the time averaging before the voxel‐wise minimum and maximum operations are performed. Therefore, the 4DRobInd might be overly conservative. In the 4DNom strategy, the time spent in each breathing phase is also not considered. For the MedEnv_05, the 4DRobAvg results were generally lower than the 4DNom results (Figure 4), showing that the motion of the tumor had a larger influence than setup and range errors, and that the dose is overestimated when not accounting for the fact that the dose in each breathing phase only contributes partly to the total dose. On the other hand, for the CTVp, 4DNom showed higher D95%. This simple evaluation could, therefore, lead to a false security that the tumor was well covered on the phases.

In most cases, only small adjustments were needed after deforming the GTVp from the reference phase to the other 4DCT phases, as seen by the high Dice score (Figure S4, (SM)). Furthermore, the differences for the DVH parameters before and after warping the dose distributions from the individual phases of the 4DCT to the reference phase were also small. We, therefore, deemed the deformable image registrations created in RayStation to be sufficiently accurate for use in the 4DRobAvg evaluation strategy. To make sure that the dose deformation is also justifiable for future clinical patients, we implemented two tests of the deformation vector field and the dose deformation, respectively. When adjusting the deformed CTVp contours, a copy of the unadjusted contours is kept, and it is required that the unadjusted CTVp contours are within the ITVp_02 contour on all phases (the ITVp is created based on the adjusted CTVp contours). Moreover, the user is notified at the end of the execution, if dose difference above 2% of the prescription dose for any of the CTV structures in any of the error scenarios and any of the phases.

Inoue et al.^25^ investigated evaluation strategies similar to the 4DNom and 4DRobAvg presented in this study. However, they only presented results for patients with a tumor motion up to 6 mm; in our study, we included also larger tumor motions up to 26 mm. Including more setup and range errors, as performed by Inoue et al., will give a more realistic scenario; however, it also increases the evaluation time. Moreover, in the study of Inoue et al., the error scenarios were combined into a scenario‐wise worst‐case dose distribution, whereas we computed voxel‐wise worst‐case dose distributions, since we aimed to develop a full 4DCT‐based evaluation method which was in line with our 3D robust evaluation strategy.^24^

Ribeiro et al. recently proposed a very elaborate and complete 4D evaluation strategy.^17^, ^22^ In their evaluation method, the interplay effect was also included by the use of machine log files to split the spots into the corresponding breathing phases of the 4DCT. They furthermore incorporated the evaluation of anatomical changes over the course of treatment by including the repeat CTs. They found the effect of anatomy changes to have the largest impact.^22^ The aim of our evaluation strategy was to assess the effect of breathing motion on the planned dose distribution to have an evaluation method before the start of treatment to judge if the plan is acceptable. Repeat CTs can, therefore, not be included at that stage of the process.

Several authors have suggested a dose evaluation strategy similar to the one presented by Ribeiro et al.,^17^ where beam delivery characteristics are considered by splitting the spots into the appropriate phase of the 4DCT, though mainly without the inclusion of repeat CTs.^5^, ^7^, ^25^, ^26^, ^27^, ^28^ Most studies simply simulate the beam delivery pattern based on assumed standard settings for the energy layer switching times and in‐plane sweeping times, whereas some use the machine log files to extract the actual spot delivery times. Moreover, the breathing pattern of the patient was either seen as constant by using a fixed breathing period for all patients (e.g., 4 s^5^ or 5 s^17^) or by applying the patient‐specific breathing period extracted from the breathing signal recorded during 4DCT acquisition^28^ or even during the daily treatments.^29^ However, Ribeiro et al.^17^ and Shan et al.^28^ also take robustness into account in combination with interplay evaluation performed by the spot splitting into subplans. Also confirmed in this study, it is important to include robustness in the dose evaluation.

It is a limitation of the current study that we did not investigate the influence of the combination of the proton spot delivery pattern and the breathing pattern on the delivered dose in a similar way as these other studies. The reason for not including such an evaluation in this study was that we prioritized an evaluation strategy as simple as possible that did not increase the computation time extensively, to ensure that the 4DRobAvg evaluation can be performed for all clinical patients with a large tumor motion. A framework to estimate the interplay effects in a specific patient in clinical practice requires a spot‐splitting workflow introducing extra computation time to setting up new plans on all the 4DCT phases (see Figure 1 by Zeng et al.^26^). A simple re‐computation is not enough due to the reduced number of spots on each phase. The 4DRobAvg evaluation presented in this study generally took less than an hour to run, which is feasible in daily clinical routine for each patient.

Our choice of not including an interplay evaluation was to some extend (but not fully) justified by the fact that we have a fractionated treatment with up to 30 fractions, which lowers the impact of the interplay effect.^5^ In addition, the large spot size of our proton delivery system^16^ is beneficial, as Grassberger et al. showed that increasing the spot size decreases the influence of the interplay effect, especially for fractionated treatment.^15^ A typical energy switching time simulated in interplay evaluation studies is ~1 s. However, our treatment machine has an energy switching time of ~50 ms,^16^ which will further reduce the impact of the interplay effect. Therefore, evaluation of the effect of the interplay between large tumor movement and the spot delivery pattern is outside of scope of this study, but will be investigated in a future work.

Another potentially important uncertainty to consider in proton treatment to moving tumors is potential changes in the motion amplitude over the course of treatment. Otter et al. found that tumor amplitude changes were largest for lung patients with large tumor amplitudes, and they found changes in the amplitude up to 8.8 mm.^30^

Applying a VW evaluation is a bit conservative since this evaluation is not based on a realistic error scenario, but instead the lowest (VWmin) or highest (VWmax) dose value in each voxel. The VWmin/VWmax dose distributions can, therefore, result in a physically unrealizable dose distribution. However, in order to use the worst scenario, to have a physically sound evaluation, an approach to rank the scenarios is needed. This can be slightly complicated considering that the lowest target dose and highest OAR dose might not occur in the same error scenario, especially not for all the OARs at once. Sterpin et al. have proposed a method to overcome this by using the value of the objective function in the optimization to rank the error scenarios.^31^

Based on the results of this study, we have started to treat lung cancer patients with a tumor movement up to 20 mm clinically. To include patients with an even larger tumor movement or an overlap between the ITVp and the diaphragm on one or more of the 4DCT (currently exclusion criteria), we are investigating the possibility to treat in breath hold.^32^ Other studies have also investigated the use of the breath‐hold technique for the treatment of lung tumors.^12^, ^33^ The advances of the breath hold technique include a reduction of dose to the OARs, which could lead to a lower risk of side effects.^34^, ^35^ These advantages could further improve the proton treatment of lung tumors with large movement, and potentially enable proton therapy for more patients, but the complexity of breath‐hold treatments is also much higher than the free‐breathing treatments investigated in this study, and moreover it is based on patient compliance.^12^

In this study, we investigated everything based on our proton delivery system from Mevion. The characteristics of this system compared to other proton therapy systems are the fairly large spot size. The results found in this study might therefore not automatically apply for other proton centers with other proton delivery systems.

## 5 CONCLUSION

We proposed an ITV‐based planning strategy on the avgCT for lung cancer patients with a tumor movement larger than 5 mm. This treatment planning strategy was found to be a clinically feasible approach which led to acceptable plans for all patients despite large tumor movement. We also developed a new 4D robust evaluation strategy to evaluate the dose coverage of the tumor in all phases of the breathing cycling. We showed that the underlying assumptions in the 4DRobAvg evaluation were justifiable and the results gave a good estimate of the dose distribution during the breathing cycle. After successful completion of this study, we have implemented the proposed treatment planning and 4D robust evaluation strategy at our proton center.

## CONFLICTS OF INTERESTS

The authors have no conflicts to disclose.

## Supporting information

Fig S1‐S5

## Data Availability

The data that support the findings of this study are available on request from the corresponding author. The data are not publicly available due to privacy or ethical restrictions.
