# Supplementary material for: Treatment planning and 4D robust evaluation strategy for proton therapy of lung tumors with large motion amplitude
Source: Med Phys. 2021 Jul 17;48(8):4425–37. doi: 10.1002/mp.15067 (PMC8456954; doi:10.1002/mp.15067)
Supplement: Supplementary file 1 — Fig S1‐S5 [file MP-48-4425-s001.pdf]

Supplemental Material

Treatment planning and 4D robust evaluation strategy for proton therapy of lung tumors with large motion amplitude

Vicki Trier Taasti, Djoya Hattu, Femke Vaassen, Richard Canters, Marije Velders, Jolein Mannens, Judith van Loon, Ilaria Rinaldi, Mirko Unipan, Wouter van Elmpt

Department of Radiation Oncology (MAASTRO), GROW – School for Oncology, Maastricht University Medical Centre+, Maastricht, Netherlands

S1. Effect of rigid transfer of delineations

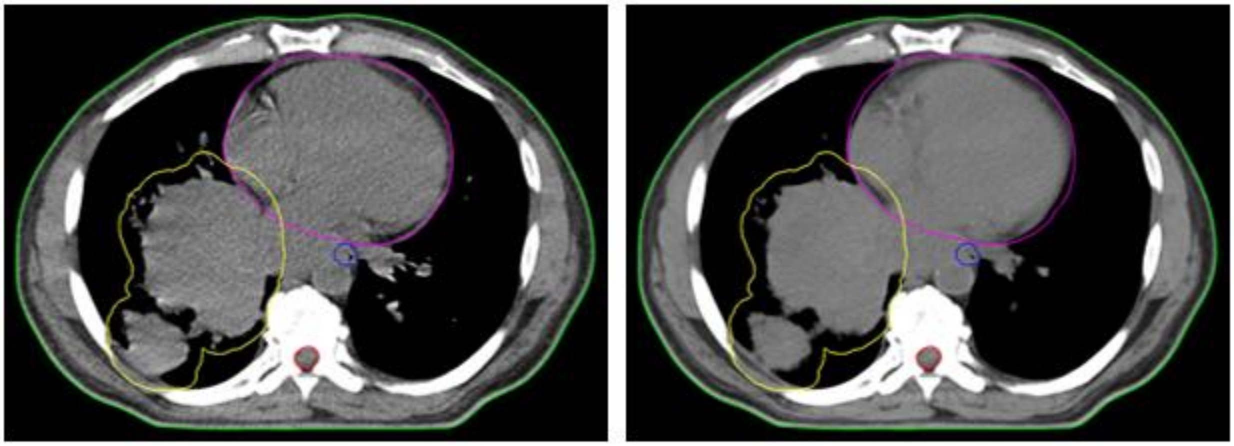

Figure S1: (Left) The CT 50% expiration phase. (Right) The average CT. The ITV is delineated in yellow in the left lower part of the images, the heart is marked in purple, the esophagus in blue, and the spinal cord in red.

It can be seen from Figure S1 that the rigid copy of the structures from the CT 50% expiration phase (left) to the average CT (right) does not cause misplacement of the structures on the average CT, which is used for plan optimization. The heart appears a bit smaller on the average CT than on the CT50ex for this patient, which is the reason for delineating on the CT50ex instead of directly on the average CT which is more blurred.

S2. Breathing patterns based on cos and $\cos^4$

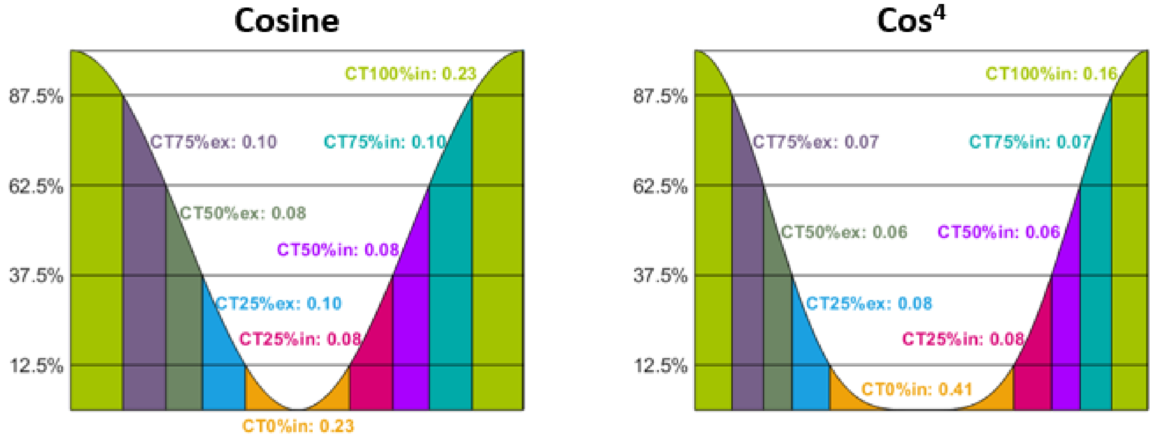

Figure S2: The weights used for the cos and $\cos^4$ breathing patterns. Each 4DCT phase is marked with a specific color. Note that the 100% inspiration phase, CT100%in, is split in a part on the left and right side of each subplot. The numbers stated in the figures are the weights used for each phase, and they correspond to the fraction of the breathing period spend in the given phase.

S3. Volume of the primary internal target volume (ITVp)

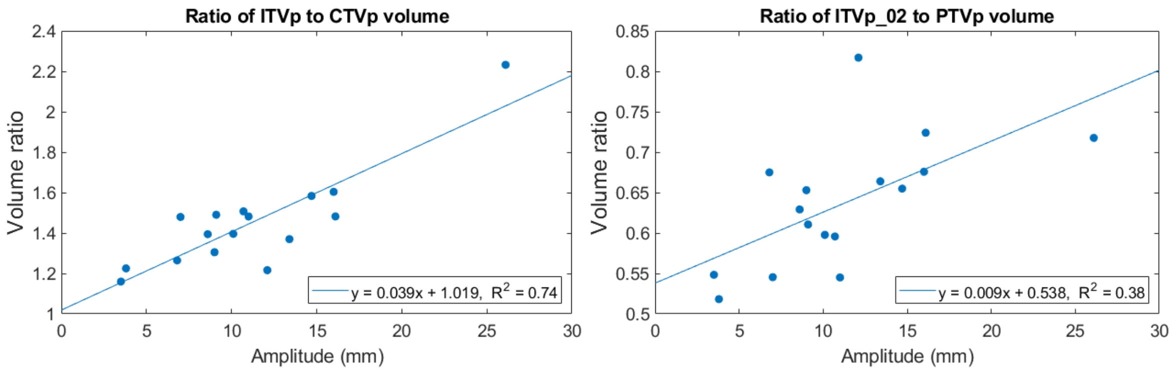

Figure S3: (Left) Ratio of the average of the CTVp volumes over the eight phases vs the ITVp volume on the average CT (planning CT), as a function of the tumor amplitude (see Table 1 in the main text for the individual data). (Right) Ratio of the ITVp_02 (ITVp expanded isotropically by 2 mm) volume on the average CT vs the PTVp volume used for the non-robust photon plan optimization as a function of the tumor amplitude.

In the left panel of Figure S3, we see a linear increase in the ratio between the volume of the primary CTV (CTVp) and the corresponding ITVp as a function of the amplitude. This is to be expected considering that the distance between the individual CTVp's on each of the eight phases would increase when the tumor amplitude is larger. On the right panel of Figure S3, the ratio between the ITVp_02 volume and the PTVp

volume is plotted again as a function of the amplitude. The ITVp_02 is used as the optimization structure in the robust proton plan optimization performed in this study, while PTVp is the optimization structure for the non-robust photon plan optimization performed in the clinic for these patients (which were all treated with photon radiotherapy). We see that for small amplitudes, the ITVp_02 is only around half the size of the PTVp. The reason for this is that in robust optimization extra dose needs to be placed around the ITVp1_02 volume to ensure good coverage, whereas in a non-robust optimization this margin is included in the PTV. As the amplitude increases the ratio of the two volumes increases towards 1. The explanation for this is that the tumor movement is directly considered in the ITVp (and thereby ITVp_02) creation, but in the creation of PTVp only the CTVp on the CT50ex is considered and an asymmetric margin in the direction of motion is then applied.

S4. Dice scores for unadjusted vs adjusted GTVp contours on the non-reference phases

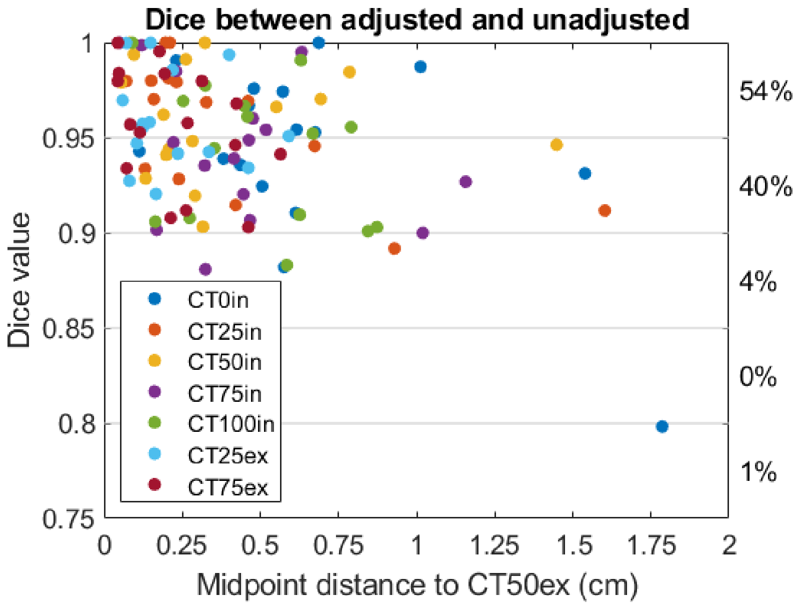

Figure S4: Dice scores for the comparison of the unadjusted and adjusted GTVp contours on the non-reference phases as a function of the distance between the GTVp mass midpoint on the reference phase (CT50ex) and on the specific the non-reference phase.

S5. Dose differences before and after deforming

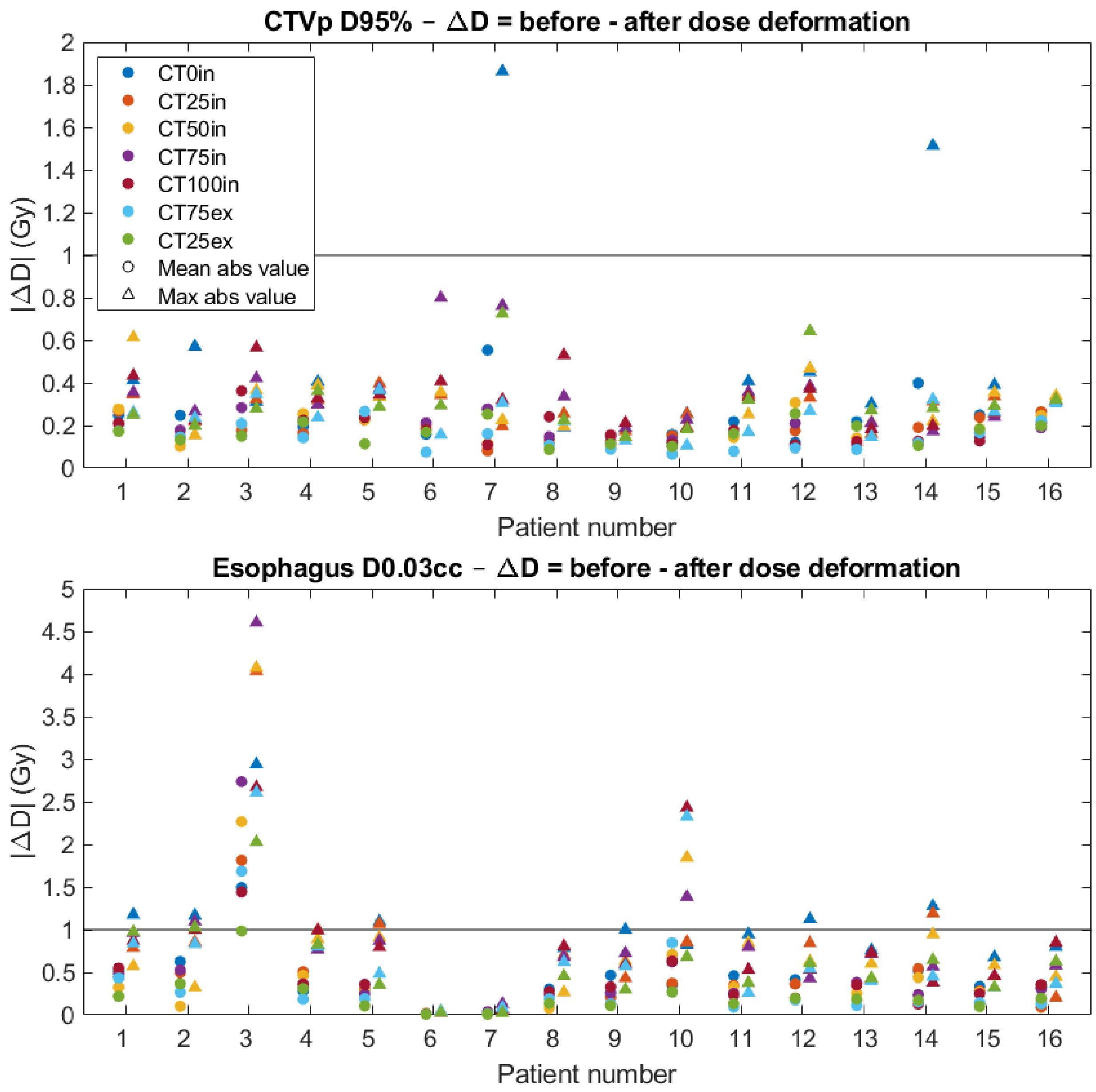

Figure S5: Dose deformation test: Absolute DVH difference before and after deforming the dose from the non-reference phase to the reference phase. (Top) D95% for the CTVp, (Bottom) D0.03cc (specifying the maximum dose) for the esophagus (DVH parameter with the largest differences in this evaluation. The legend in the top image corresponds to both subplots. The circles show the mean DVH difference over the 28 scenarios for each of the seven phases, and the triangles show the maximum DVH difference. The threshold of 1 Gy (vertical line) was chosen arbitrarily.
